# Editorial: Endothelium, innate immunity and coagulation in hematological disorders

**DOI:** 10.3389/fmed.2026.1854580

**Published:** 2026-05-04

**Authors:** Paschalis Evangelidis, Eleni Gavriilaki

**Affiliations:** 1 Second Propedeutic Department of Internal Medicine, Hippocration Hospital, Aristotle University of Thessaloniki, Thessaloniki, Greece; 2 Transplantation Unit, Hematology Department, Endothelial Injury Excellence Centre, Bone Marrow G. Papanicolaou General Hospital of Thessaloniki, Thessaloniki, Greece

**Keywords:** coagulation, complement, endothelium, hematological disorders, innate immunity, thromboinflammation

In recent years, the interplay between endothelium, innate immunity, and coagulation has emerged as one of the most active research areas in hematology (1, 2). Once considered as separate processes, endothelial injury, inflammatory signaling, complement dysregulation, platelet activation, and thrombin generation are now recognized as tightly interconnected contributors to disease pathogenesis across several hematologic conditions (3). This fact is particularly important in disorders in which thrombosis and bleeding coexist, in complement-mediated syndromes, in hematopoietic cell transplantation-related complications, and in immune-mediated toxicities following cellular therapies (4, 5).

Therefore, we organized this Research Topic aiming to bring together translational, diagnostic, and clinical studies addressing this evolving triad in hematological disorders. The 25 contributions collected present novel insights concerning thrombotic microangiopathies (TMAs), venous thromboembolism, immune cytopenias, bone marrow failure syndromes, hematologic malignancies, chimeric antigen receptor-T (CAR-T) cell toxicities, supportive transfusion care, and even physiologic hematologic adaptation to chronic hypoxia. All together, these original clinical and translational studies, case reports, and review articles share a common “message”: endothelial injury, innate immune activation, and coagulation imbalance are not isolated events, but constitute overlapping biologic mechanisms that drive disease manifestations, contribute to organ injury, and increasingly inform the development of novel targeted treatments.

## TMAs and complement-mediated endothelial injury

Few disease groups illustrate the coexistence of endothelial injury, complement, and coagulation activation more clearly than TMAs. In these disorders, microvascular endothelial damage, platelet-mediated thrombosis, hemolysis, and organ injury are linked through well-recognized pathways involving complement, von Willebrand factor processing, inflammatory triggers, and vascular injury (6, 7).

Huang et al. report a clinically instructive case of congenital thrombotic thrombocytopenic purpura (cTTP) caused by a compound heterozygous mutation in the ADAMTS13 (a disintegrin and metalloproteinase with thrombospondin type 1 motif, member 13) gene in a young adult who was initially misdiagnosed with immune thrombocytopenia (ITP). Beyond emphasizing the importance of early ADAMTS13 testing and genetic evaluation in recurrent thrombocytopenia with co-existing organ injury, this case report is notable for the observation of elevated soluble C5b-9 (sC5b-9) in the absence of detectable complement gene mutations, supporting the concept that complement activation may amplify endothelial injury even in TTP, classically defined by severe ADAMTS13 deficiency.

A similarly important “complement-centered” perspective is provided by Jodele and Gavriilaki, who review the translation of biomarker insights into clinical practice in transplant-associated TMA. Their work highlights how longitudinal biomarker evaluation, particularly of sC5b-9, proteinuria, hemolysis parameters, and organ injury features, can improve diagnosis, refine risk stratification, and even guide treatment selection.

The clinical and biologic heterogeneity of complement-mediated TMA, also described as atypical hemolytic uremic syndrome (aHUS), is further illustrated by Wu et al., who report a pregnancy-associated aHUS case in a patient with thalassemia and a complement factor H mutation. Specifically, they describe a “two-hit” hypothesis implicated in the pathogenesis of aHUS in this patient: complement dysregulation due to inherited susceptibility may be intensified by hemolysis-related amplification of the complement cascade, due to co-existing thalassemia, with potential implications for response to C5 inhibition (8). Similarly, Gao et al. present a case of paroxysmal nocturnal hemoglobinuria, initially masquerading as aHUS, reminding us that intravascular hemolysis, renal injury, and thrombosis often blur traditional diagnostic boundaries and demand careful differential diagnosis (9, 10).

**Figure 1:**
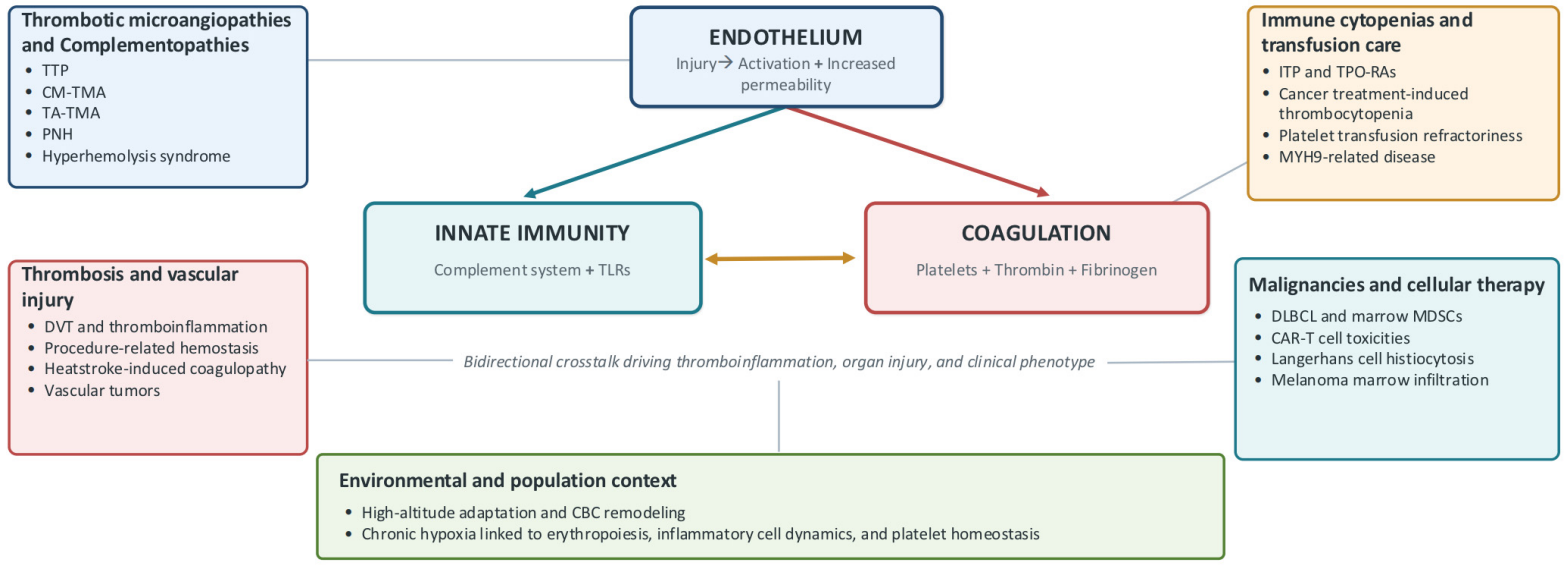
The endothelium-innate immunity-coagulation axis across hematological disorders. CAR-T, chimeric antigen receptor T-cell; CBC, complete blood count; CM-TMA, complement-mediated thrombotic microangiopathy; DLBCL, diffuse large B-cell lymphoma; DVT, deep vein thrombosis; ITP, immune thrombocytopenia; MDSCs, myeloid-derived suppressor cells; MYH9, myosin heavy chain; PNH, paroxysmal nocturnal hemoglobinuria; TA-TMA, transplant-associated thrombotic microangiopathy; TLRs, toll-like receptors; TPO-RAs, thrombopoietin receptor agonists; TTP, thrombotic thrombocytopenic purpura.

Together, these contributions further reinforce the idea that TMAs are not as isolated clinical entities but constitute syndromes positioned along a continuum of endothelial injury, complement activation, and microvascular thrombosis (Figure 1), also highlighting how novel diagnostic methods increasingly depend on integrating classical hematologic parameters, along with molecular testing, endothelial biomarkers, and organ-specific assessment.

## Thromboinflammation in thrombosis and vascular injury

The reciprocal relationship between coagulation and inflammation is a recurring theme throughout this Research Topic. Deep vein thrombosis (DVT), in particular, historically described as a disorder of blood stasis and hypercoagulability, is now understood as a thromboinflammatory process in which endothelial dysfunction, leukocyte recruitment, platelet activation, and innate immune signaling are deeply connected.

Kahraman et al. address this concept directly in their study on D-dimer and the systemic inflammation response index (SIRI) in DVT. Their findings support the diagnostic value of inflammatory indices derived from routine laboratory testing and show that combining SIRI with D-dimer improves predictive performance for DVT identification. In parallel, Shao, Wang, Wu et al. review the role of toll-like receptors in DVT and discuss their therapeutic potential. By focusing on platelet activation, endothelial dysfunction, leukocyte recruitment, and danger-associated signaling, they provide a clear mechanistic framework for understanding how innate immune pathways contribute to venous thrombosis, and potentially in other cardiovascular disorders (11).

Other contributions broaden this thromboinflammatory perspective beyond classical venous thrombosis. Yao et al. review anticoagulant regimens across different therapeutic plasma exchange modalities, a clinically relevant topic in several hematological disorders, such as TTP, and non-hematological clinical syndromes. Their review adds practical clinical guidance to this Research Topic.

Shin et al. identify in their study activated clotting time as a predictor of incomplete hemostasis after transradial coronary intervention, associating procedural hemostatic outcomes with measurable coagulation dynamics. Furthermore, Zheng et al. found that thromboelastography can sensitively track perioperative coagulation changes in patients with hepatic echinococcosis, highlighting the value of whole-blood functional testing in complex inflammatory and surgical settings. Xiang et al., in their review of heatstroke-induced coagulopathy, discuss how systemic inflammation, endothelial injury, and coagulation failure can evolve into either hypo- or hypercoagulable states, underlining that this hemostatic imbalance is inseparable from innate immune activation.

Two additional case reports concerning vascular pathologies are presented. Shao, Wang, Wang et al. describe epithelioid hemangioendothelioma of the femoral artery initially mistaken for arterial occlusion, while Liu et al. report malignant melanoma with bone marrow infiltration leading to coagulation dysfunction and spinal epidural hematoma. Although distinct in biology, both cases show how disruption of vascular integrity and tumor-associated coagulopathy can lead to severe clinical presentations, delayed diagnosis, and poor outcomes.

## Immune cytopenias, hemolysis, and platelet-directed care

Several articles in this Topic focus on thrombocytopenia, hemolysis, and supportive hemostatic care, showing how coagulation, inflammation, complement activation, and vascular injury are present in various hematological disorders not classically described as thrombotic diseases.

Luo et al., in their systematic review and meta-analysis, bridge randomized clinical trials (RCT) and real-world studies of thrombopoietin receptor agonists in adult primary ITP. Moreover, Zhao et al. review cancer treatment-induced thrombocytopenia, a common and clinically challenging complication that affects bleeding risk, treatment continuity, and quality of life in these patients. In addition, Zou et al. developed a predictive model for platelet transfusion refractoriness in hematological patients, identifying clinically relevant risk factors that may support personalized transfusion strategies in clinical practice.

Lian et al. describe a family with MYH9 (myosin heavy chain 9)-related disease initially misdiagnosed as ITP, emphasizing the importance of peripheral blood morphology, family history, and genetic testing in cases of isolated thrombocytopenia.

The overlap between hemolysis, inflammation, and thrombosis is also well-illustrated by Wang, Li et al., who report a case of mixed-type autoimmune hemolytic anemia complicated by acute cerebral infarction. Complement blockade, nowadays, emerges as a novel treatment option for patients with autoimmune hemolytic anemia (13). Aqel et al. extend this hemolysis-centered discussion through their review of hyperhemolysis syndrome, focusing on the use of eculizumab and tocilizumab in refractory cases.

Furthermore, Shen and Wei, in their RCT, evaluate the impact of quality nursing and health education in severe aplastic anemia patients complicated by infection, with an emphasis on inflammatory indices, immune recovery, and clinically meaningful outcomes.

## Hematologic malignancies, bone marrow niche, and cellular therapy toxicities

Efstratiou et al. showed that myeloid-derived suppressor cells (MDSCs) in diffuse large B-cell lymphoma are expanded in both blood and bone marrow, with marrow-derived monocytic MDSCs displaying a distinct transcriptional profile. This work enhances our understanding of innate immune remodeling in lymphoma, suggesting that the bone marrow niche is not merely a passive reservoir but an active compartment of immune suppression that may influence therapeutic resistance.

The interplay between immune activation and endothelial injury becomes even more apparent in toxicities following CAR-T cell therapy (12). Moreno-Castaño et al. review endothelial dysfunction and hemostatic imbalance in CAR-T-associated toxicities, with particular emphasis on circulating biomarkers that may predict cytokine release syndrome, neurotoxicity, coagulopathy, and hemophagocytic inflammation. Additionally, a real-world study is provided by our group, reporting management strategies for CAR-T-related toxicities across six transplant centers in Greece (Gavriilaki et al.). Considered together, these two contributions show how a mechanistic understanding of endothelial injury can inform safer implementation of cellular therapies in routine care.

Zeng et al. present an adult-onset multisystem Langerhans cell histiocytosis case initially manifesting as arginine vasopressin deficiency, emphasizing the need for diagnostic vigilance in rare hematopoietic disorders. Liu et al., as discussed above, demonstrate how marrow infiltration by melanoma can drive severe coagulopathy and neurologic complications.

## High-altitude adaptation and blood count interpretation

Wang, Liu et al. establish altitude-, sex-, and age-specific complete blood count reference intervals in healthy adults from the Western Sichuan Plateau and show that increasing altitude is associated with higher red blood cell, hemoglobin, hematocrit, white blood cell, and platelet values. The importance of this work lies not only in improving local diagnostic accuracy but also in illustrating how chronic hypoxia remodels hematologic physiology in ways that may influence endothelial stress, inflammatory signaling, and platelet homeostasis. Abdulrasak et al., in their commentary on this study, extend the discussion by emphasizing the potential relevance of ancestry, regulatory variants, and population-specific diagnostics.

## Conclusions

In summary, the articles in this Research Topic highlight endothelial injury, innate immunity, and coagulation as tightly interconnected determinants of disease phenotype, organ injury, and clinical outcome across hematologic disorders (Figure 1). Overall, this Research Topic supports a more unified view of hematologic diseases as a dynamic interplay of endothelial stress, immune activation, and hemostatic imbalance, with important implications for diagnosis, risk stratification, and treatment. Future research agenda in the field should include:

- Validation of biomarkers of endothelial injury, inflammation, complement activation, and coagulation across hematologic disorders.
- Development of biomarker-guided treatment strategies, especially for complement inhibition and cellular therapy toxicities.
- Application of machine learning to multimodal data to improve diagnosis, risk stratification, and prediction of treatment response across thromboinflammatory hematologic disorders.
- Integration of patient-reported outcomes into future studies to better capture symptom burden, quality of life, and long-term functional impact beyond laboratory and survival endpoints.
